# Anesthetic considerations in Demons-Meigs’ syndrome: a case report

**DOI:** 10.1186/1752-1947-8-320

**Published:** 2014-09-27

**Authors:** Salaheddine Fjouji, Mustapha Bensghir, Charki Haimeur, Hicham Azendour

**Affiliations:** 1 Department of Anaesthesiology, Military Hospital Med V Rabat, University of Med V Souissi, Avenue des Nations Unies, Rabat 10000, Morocco

**Keywords:** Demons-Meigs’ syndrome, Anesthesia, Ascites, Pleural effusion, Abdominal hypertension

## Abstract

**Introduction:**

Demons-Meigs’ syndrome is characterized by the presence of a benign ovarian tumor associated with ascites and a right-sided hydrothorax. Its pathophysiology remains unclear. Anesthesia of this syndrome is a real challenge. Respiratory, hemodynamic, metabolic problems and abdominal hypertension are the main anesthetic risks.

**Case presentation:**

A 52-year-old African woman with Demons-Meigs’ syndrome was admitted for elective surgery under general anesthesia. An abdominal computed tomography scan showed a tumor mass, with tissue and cystic components associated with abundant ascites and a right pleural effusion of medium abundance. In the operating room after standard monitoring, a crash induction was performed. Just after, her saturation level decreased requiring the use of an alveolar recruitment maneuver followed by the application of positive end-expiratory pressure. Vasoconstrictor and vascular filling were used to correct the hypotension that occurred. Airway pressures remained at 35cm H2O. Maintenance of a slightly proclive position and opening of the abdomen with the progressive removal of 3200ml ascitic fluid allowed a lower thoracic pressure (airway pressures=24cm H2O). Her postoperative course was unremarkable. Clinical evolution after five months was marked by a complete recovery of our patient and no recurrence of effusion or ascites.

**Conclusions:**

Demons-Meigs’ syndrome is a benign disease with a good prognosis. Respiratory and hemodynamic problems and abdominal hypertension are the main anesthetic risks of this syndrome. Good management of these risks is necessary to preserve the prognosis.

## Introduction

Demons-Meigs’ syndrome is characterized by the presence of a benign ovarian tumor associated with ascites and a right-sided hydrothorax [1,2].

It is rarely seen and its pathophysiology remains unclear [3,4]. Differential diagnosis with ovarian neoplasia should be discussed before surgery. The pleural effusion and ascites resolve spontaneously and permanently after removal of the tumor [5].

Anesthesia of this syndrome is a real challenge. Respiratory, hemodynamic and metabolic problems and abdominal hypertension are the main anesthetic risks [6-8]. Management of these risks is a perioperative priority. The authors present a clinical case involving the anesthetic management of this syndrome and a review of the literature.

## Case presentation

**Figure 1:**
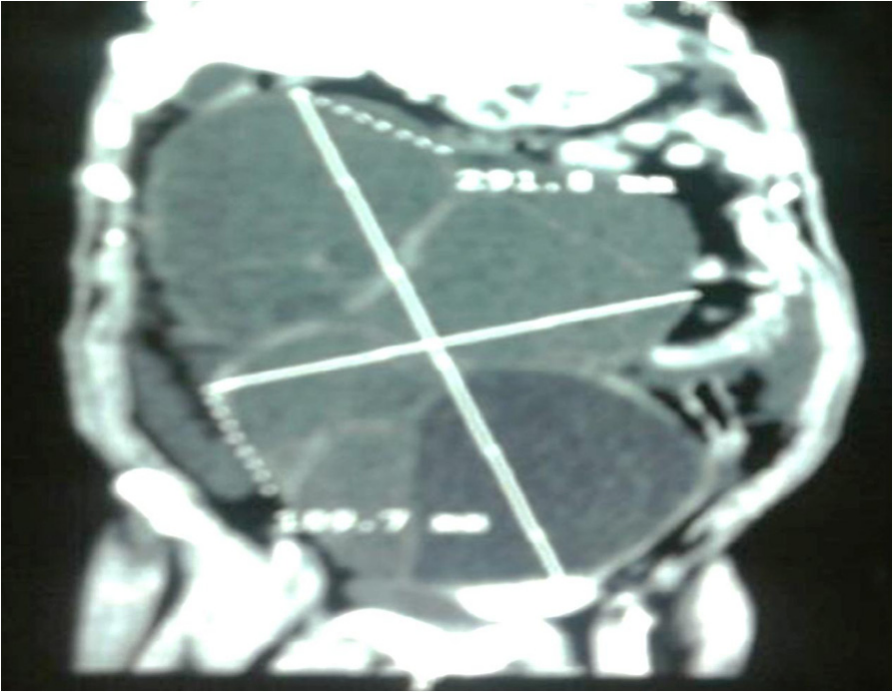
Computed tomography scan showing a huge tumor with tissue and cystic components.

**Figure 2:**
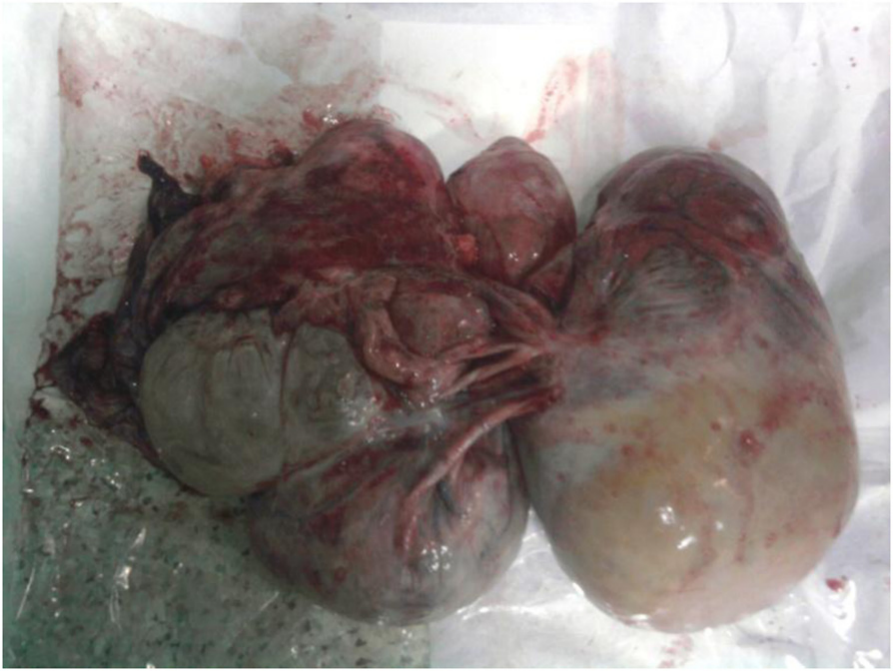
Abdominal tumor encapsulated after resection surgery.

A 52-year-old African woman with primary infertility who had been postmenopausal for eight years presented with abdominal heaviness of nine months’ duration recently associated with postprandial vomiting. No change of her general state was noted. There were no signs of compression of the pelvic organs. Abdominal ultrasonography showed a huge abdominal mass and an effusion of average abundance. An abdominal computed tomography (CT) scan (Figure 1) showed a tumor mass, tissue and a cystic component measuring 189×133×291mm displacing the bowel loops up and out, and the bladder and uterus down, associated by abundant ascites and a right pleural effusion of medium abundance. The level of carbohydrate antigen 125 (CA-125) was 133U/ml. Surgical exploration was indicated. A preoperative examination noted NYHA (New York Heart Association) class II dyspnea, and obesity with a body mass index (BMI) of 38.6kg/m^2^. An examination of her upper airway noted a Mallampati grade II. Auscultation found silence at the base of her right lung. Her abdomen was distended. Her pulse oximetry indicated 97%. Her electrocardiogram was normal. A preoperative chest X-ray showed effusion syndrome of medium amount. Echocardiography showed good cardiac function with an ejection fraction of 68%. There were no electrolytic disorders: potassium at 4.2mmol/l, fasting plasma glucose at 1.05g/l. Her renal function was not impaired, and hemostasis laboratory tests were normal. Her preoperative hemoglobin was 12.2g/dl. Our patient was premedicated with 50mg hydroxizine the day before surgery and 50mg on the morning of the operation. In the operating room, standard monitoring (noninvasive blood pressure, scope, arterial oxygen saturation) was performed. Venous access was secured by two 16G catheters and 2g cefazolin was administered. After 10 minutes of preoxygenation in a twenty-degree reverse-Trendelenburg position, and a vascular filling of 1000ml of crystalloid, the crash induction was administered using 100mg suxamethonium, 500mg thiopental and the Sellick’s maneuver. Oral tracheal intubation was successful at the first attempt by a tube 7mm in diameter and our patient was connected to the anesthesia machine and ventilated with a tidal volume of 480ml, respiratory rate of 14 cycles/minute. Just after connecting our patient to the ventilator, her saturation level decreased to 96%. To treat and prevent a secondary drop in saturation, an alveolar recruitment maneuver was decided upon. This maneuver was performed by the application of continuous positive airway pressure (40cm H2O/40s). After this maneuver, her positive end-expiratory pressure (PEEP) was maintained at 8cmH20. At the end of this recruitment procedure, her blood pressure decreased to 86/51mmHg, requiring filling with 500ml crystalloid and two boluses of ephedrine (60mg) to achieve a blood pressure of 112/65mmHg. After respiratory and hemodynamic stabilization, maintenance of anesthesia was provided by a mixture of oxygen, nitrous oxide (60%:40%) and 2% of sevoflurane. Airway pressures (Paw) remained at 35cm H2O with a saturation level of 99% under a fraction of inspired oxygen at 60% and PEEP of 8cmH20. Maintenance of the slightly proclive position and opening of the abdomen with the progressive removal of 3200ml ascitic fluid allowed a lower thoracic pressure (Paw=24cm H2O). Her hemodynamic status remained stable with a filling of 1000ml of saline serum (0.9%). Intraoperative bleeding was estimated at 600ml, her hemoglobin at the end of surgery was 10.9g/dl and no operative transfusion was administered. The surgical procedure consisted of resection of the tumor, hysterectomy, and omentectomy (Figure 2). Her bladder and bowel loops were compressed without evidence of invasion. The surgical intervention required 3 hours and 30 minutes. Diuresis at the end of the surgery was to 550ml. The intraoperative analgesia was provided by paracetamol (1g), nefopam (20mg) and morphine (5mg) by slow infusion. This analgesia was relayed postoperatively by paracetamol 1g/6h, nefopam 20mg/8h, and morphine patient-controlled analgesia (PCA). In the recovery room, extubation was made after warming, wakening and stabilizing of her respiratory and hemodynamic parameters. The monitor showed a blood pressure of 145/68mmHg and blood oxygen saturation (SpO2) of 98%. Our patient remained on oxygen for 2 hours under 4L/min of flow. Postoperative chest radiography showed no worsening of the effusion. Her hemoglobin and postoperative blood electrolytes were unremarkable. Prevention of thromboembolic disease was started 6 h after the end of the surgery based on enoxaparine 40mg/24h and compression stockings. The nature of the tumor histology was thecoma with a benign prognosis, confirming Demons-Meigs’ syndrome. Clinical evolution after five months was marked by a complete recovery of our patient and no recurrence of effusion.

## Discussion

Demons-Meigs’ syndrome can cause severe respiratory and hemodynamic complications during anesthesia caused by a giant mass in the peritoneal space, massive ascites and pleural effusion. Theories to explain the pathophysiology of the peritoneal and pleural effusion have been proposed. Mechanical compression or partial torsion of fibromas or other benign ovarian solid tumors leads to the ascites production by the lymph released from the surface of the tumor [9]. There is exudation from the peritoneum because of mechanical irritation by the heavy mobile tumor [2]. There is injury or necrosis of cyst formations within the tumor [10]. Recently, a probable active secretion by the tumor of the growth factors mediating the hyperpermeability in the ovary or the peritoneal vessel was suggested [11]. In the mechanism of hydrothorax production, ascites are believed to migrate to the right thoracic cavity through a congenital defect or overloaded lymphatics, which are more common on the right [12]. Hence, there is a high incidence of right-sided hydrothorax.

Patients with Demons-Meigs’ syndrome complain of abdominal and respiratory symptoms [6]. The increased intraperitoneal pressure, caused by the contents in the peritoneal cavity, can have adverse physiologic effects, such as decreased cardiac output, ventilation-perfusion abnormalities with an accompanying hypoxia and hypercapnia, reduction in renal perfusion flow and glomerular filtration with decreased urine output, elevation in intracranial pressure, and impaired liver and gastrointestinal perfusion with digestive transit disorders. But the true challenge for anesthetists in this syndrome is the abdominal hypertension and its potential evolution to abdominal compartment syndrome [8,13-15]. The intra-abdominal hypertension is defined by a sustained or repeated pathological elevation in intra-abdominal pressure ≥12mmHg. Evolution can be to abdominal compartment syndrome, which is defined as a sustained intra-abdominal pressure >20mmHg that is associated with new organ dysfunction/failure [16].

Different cases of abdominal compartment syndrome complicating Demons-Meigs’ syndrome have been reported [8,13-15]. Treatment of this syndrome is based on vascular filling, sedation, analgesia, the reverse Trendelenberg position, neuromuscular blockade and surgical decompression [16]. The indications for this decompression remain controversial. In Demons-Meigs’ syndrome, complicated by intra-abdominal hypertension, surgical indication is retained for excision of the abdominal mass even in the absence of intra-abdominal hypertension [8]. In preoperative preparation, measurement of intra-abdominal pressure is made especially to graduate and monitor intra-abdominal hypertension. In the postoperative period, this measurement is necessary in case of massive filling or bleeding with the establishment of packing. In our patient, a measurement of intra-abdominal pressure was not made. Surgical indication was retained for excision of the tumor.

Many patients with Meigs’ syndrome are undernourished, with an associated anemia and electrolyte imbalance, which require correction [6]. To overcome these problems, anesthetic management starts with a good preoperative evaluation that assesses the importance of the respiratory, hemodynamic and metabolic impact. Pleural effusion may cause chest pain, shortness of breath; the quantity of liquid is evaluated by a chest X-ray. Extreme, hypoxic respiratory distress requires aspiration of the pleural and abdominal transudates to restore respiratory function and physiology to as near normal limits as possible. Aspiration should be repeated if necessary: the final aspirates being done on the day before the operation [6].

In our patient, several factors contributed to the desaturation, the abdominal compartment syndrome related to Demons-Meigs’ syndrome and the formation of atelectasis, related to anesthetic induction and especially paralysis. To correct these intraoperative desaturation problems, several procedures were used, however, alveolar recruitment maneuvers remained the most effective procedure [17-19]. The only limitation of these maneuvers is their hemodynamic consequences. In our case desaturation was deep, authorizing the use of these maneuvers. The decrease in blood pressure was corrected by volume expansion and vasoconstrictors.

Preoperative anemia is common Demons-Meigs’ syndrome [6] and is associated with increased likelihood of blood transfusion and increased perioperative morbidity. Correction by transfusion of red blood cells or whole blood, when they are necessary, is required. Other electrolytic disorders should be corrected before surgery. Premedication with hydroxizine has been shown to be safe. An opioid-based or benzodiazepine medication should be avoided because of the risk of preoperative respiratory depression, hypoventilation and hypoxemia [6]. Because of risk of regurgitation consequent of the increase in the intra-abdominal pressure and restricted diaphragmatic movement, the anesthetic technique is a crash induction with application of the Sellick’s maneuver after preoxygenation in a proclive position. Thoracic pressures are generally high, this problem can be solved by proclive positioning, rapid laparotomy to drain the ascites, and incidental use of autoflow ventilator mode. A postoperative chest X-ray to search for residual pleural effusion is indicated. It is rarely necessary to perform chest aspiration following the operation, but hypoxia during recovery has been described [3].

Excision of a huge tumor is associated with risk of bleeding due to the size of the tumor, and its anatomical relationships. It can result in hemodynamic intraoperative alteration, aggravated by the inconvenience of venous return due to abdominal pressure. This requires adequate intraoperative monitoring, performing of transfusion laboratory tests, anticipation of blood loss by optimizing blood volume and transfusion thresholds accordingly. As in any oncologic pelvic surgery, antibiotic prophylaxis is based on a first- or second-generation cephalosporin.

The management of postoperative pain is made by a multimodal analgesia and intravenous opioids. The use of epidural analgesia has been described as an efficient option in such cases [7]. Prevention of thromboembolic events is achieved by low-molecular-weight heparin combined with compression stockings and early ambulation.

## Conclusions

Demon-Meigs’ syndrome is a benign disease with a good prognosis. The respiratory and hemodynamic risks of this syndrome are the main anesthetic problems. Perioperative optimal management of these risks preserves the good prognosis of this syndrome.

## Consent

Written informed consent was obtained from the patient for publication of this manuscript and any accompanying images. A copy of the written consent is available for review by the Editor-in-Chief of this journal.

## Abbreviations

BMI: body mass index; CA: carbohydrate antigen; CT: computed tomography; NYHA: New York Heart Association; Paw: airway pressures; PCA: patient-controlled analgesia; PEEP: positive end-expiratory pressure; SpO2: blood oxygen saturation.

## Competing interests

The authors declare that they have no competing interests.

## Authors’ contributions

SF and MB analyzed and interpreted the patient data. SF and MB were major contributors in writing the manuscript. CH and HA made the final corrections. All authors read and approved the final manuscript.
